# Dexmedetomidine Reduces Presynaptic γ-Aminobutyric Acid Release and Prolongs Postsynaptic Responses in Layer 5 Pyramidal Neurons in the Primary Somatosensory Cortex of *Mice*

**DOI:** 10.3390/ijms26051931

**Published:** 2025-02-24

**Authors:** Bo Tang, Jiali Tang, Yuguang Huang

**Affiliations:** Department of Anesthesiology, Peking Union Medical College Hospital, Peking Union Medical College & Chinese Academy of Medical Sciences, Beijing 100730, China; doctortangbo@163.com

**Keywords:** dexmedetomidine, GABAergic transmission, inhibitory postsynaptic signal, somatosensory cortex, layer 5

## Abstract

Dexmedetomidine (DEX) exhibits notable sedative, analgesic, and anesthetic-sparing properties. While growing evidence suggests these effects are linked to the modulation of γ-aminobutyric acid (GABA) system, the precise pre- and postsynaptic mechanisms of DEX action on cortical GABAergic signaling remain unclear. In this study, we applied whole-cell patch-clamp recording to investigate the impact of DEX on GABAergic transmission in layer 5 pyramidal neurons of the *mouse* primary somatosensory cortex. We recorded spontaneous inhibitory postsynaptic currents (sIPSCs), miniature IPSCs (mIPSCs), and evoked inhibitory postsynaptic potentials (eIPSPs) before and during DEX application. Our findings demonstrated that DEX reduced activity-dependent spontaneous GABAergic transmission, as evidenced by a decrease in sIPSC frequency, while mIPSC frequency was unaffected. eIPSPs were not significantly influenced by DEX either. Additionally, DEX prolonged the kinetics of both sIPSCs and mIPSCs, increasing the rise and decay times of sIPSCs and the decay time of mIPSCs. We proposed that DEX modulated cortical neuronal activity by limiting GABA release and altering GABA_A_ receptor kinetics. Collectively, these results indicated that DEX modulated cortical GABAergic signaling at both presynaptic and postsynaptic sites, which likely underlined its sedative, analgesic, and anesthetic-sparing effects.

## 1. Introduction

Dexmedetomidine (DEX), a highly selective α_2_-adrenoceptor (α_2_-AR) agonist, is widely used as an anesthetic adjuvant due to its sedative and analgesic properties [1]. While the initial understanding of DEX’s mechanism of action focused on its ability to reduce noradrenaline release by decreasing the firing rate of adrenergic neurons in the locus coeruleus [2], accumulating evidence suggests that its reliable sedation, easy arousal, and cognitive-sparing effects may also involve direct actions on non-adrenergic neurons [3,4,5,6]. Indeed, previous studies indicated that DEX-mediated modulation of the γ-aminobutyric acid (GABA) system significantly affects brain functions. In subcortical areas, DEX has been proposed to induce non-rapid eye movement-like sleep by activating α_2_-ARs on inhibitory afferent axon terminals in the preoptic hypothalamic area (POA) or on GABAergic neurons within the POA [2,3]. Furthermore, DEX has been shown to reduce excessive α_5_ GABA_A_ receptor (GABA_A_R) function in hippocampal neurons, thereby mitigating cognitive deficits following general anesthesia [7]. These findings highlight the potential importance of DEX’s actions on GABAergic transmission in various brain regions. However, the specific impact of DEX on the efficacy of GABAergic transmission in the neocortex, where GABAergic circuits play critical roles in information processing [8], remains to be fully clarified. In the present study, the pre- and postsynaptic effects of DEX on cortical GABAergic transmission were investigated.

## 2. Results

### 2.1. DEX Decreased the Frequency and Slowed the Kinetics of sIPSCs

**Figure 1:**
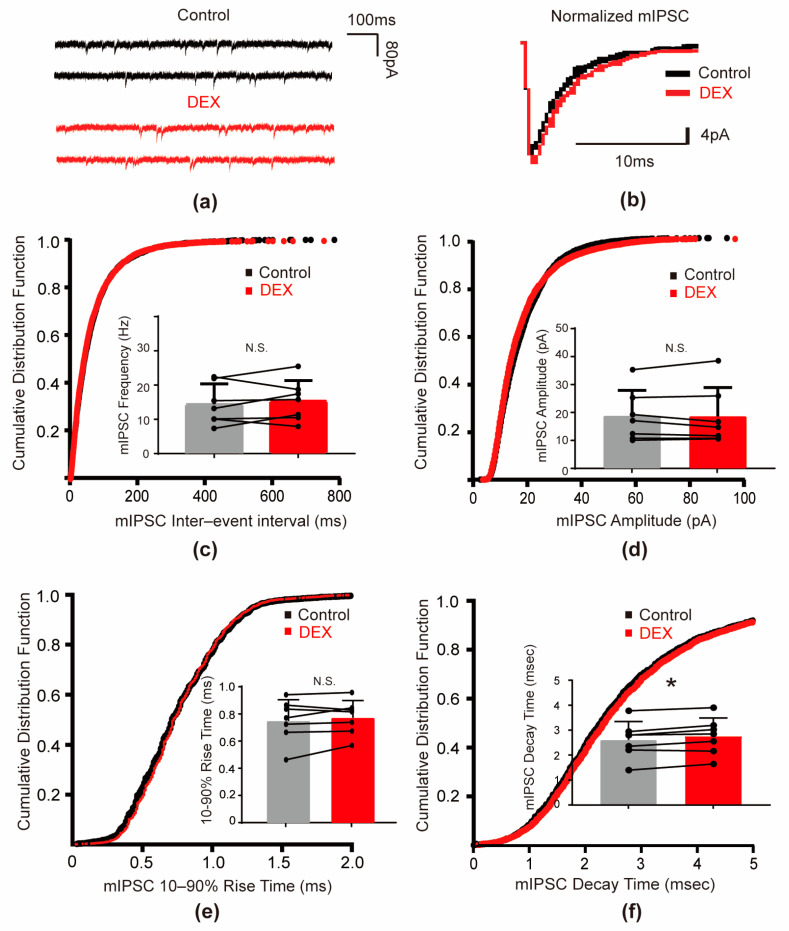
DEX decreased the frequency and slowed the kinetic parameters of sIPSCs in S1 layer 5 pyramidal cells. (**a**) Representative traces illustrating sIPSCs in the absence of DEX versus sIPSCs in the presence of DEX (10 μM); (**b**) representative traces of normalized sIPSCs in the absence of DEX versus sIPSCs in the presence of DEX (10 μM); (**c**,**d**) the cumulative probability curve of interevent intervals and the amplitudes of sIPSCs before and during DEX application. The insert bars in (**c**,**d**), summarized the effect of DEX on the frequency (*p* = 0.024) and amplitude (*p* = 0.158) of sIPSCs; (**e**,**f**) cumulative probability curve of the 10–90% rise time and decay time of sIPSCs before and during DEX application. The insert bars in (**e**,**f**) summarize the increased 10–90% rise time (*p* = 0.038) and decay time (*p* = 0.015) of sIPSCs. Dots represent the value of each cell. The asterisk (*) indicated *p* < 0.05 and N.S. indicated no significant differences (n = 9). Control indicated artificial cerebrospinal fluid (aCSF) without DEX.

To investigate the impact of DEX on spontaneous inhibitory neurotransmission, we recorded spontaneous inhibitory postsynaptic currents (sIPSCs) from layer 5 pyramidal neurons in the primary somatosensory cortex of *mouse* (S1). To isolate inhibitory currents, 6-cyano-7-nitroquinoxaline-2,3-dione (CNQX) and D-2-amino-5-phosphonovalerate (D-AP5) were used to block excitatory signals. These recordings were performed with a holding potential of −70 mV. Bath application of 10 μM DEX significantly decreased the frequency of sIPSCs. The mean sIPSC frequency decreased from 16.27 ± 10.55 Hz in control conditions to 13.78 ± 8.68 Hz after DEX treatment (a 15.30% decrease, *p* = 0.028, Figure 1c). This was further confirmed by a rightward shift in the cumulative probability curve of sIPSC interevent intervals. In contrast, DEX did not significantly alter sIPSC amplitude (control: 18.77 ± 3.65 pA versus DEX: 17.65 ± 2.77 pA, *p* = 0.139, Figure 1d). These results suggested that DEX reduced the release of GABA at presynaptic terminals.

Next, we examined whether DEX influenced the kinetics of sIPSCs. We found that DEX significantly slowed both the activation and deactivation phases of sIPSCs. The 10–90% rise time of sIPSCs increased from 0.88 ± 0.19 ms to 0.93 ± 0.17 ms (*p* = 0.038, Figure 1e), and the decay time increased from 3.18 ± 0.75 ms to 3.45 ± 0.67 ms (*p* = 0.015, Figure 1f) upon DEX application. These changes were reflected by rightward shifts in the cumulative frequency curves of the rise and decay times. These findings indicated that DEX prolonged the duration of GABAergic inhibitory currents in postsynaptic pyramidal neurons.

### 2.2. DEX Prolonged the Decay Time of mIPSCs

**Figure 2:**
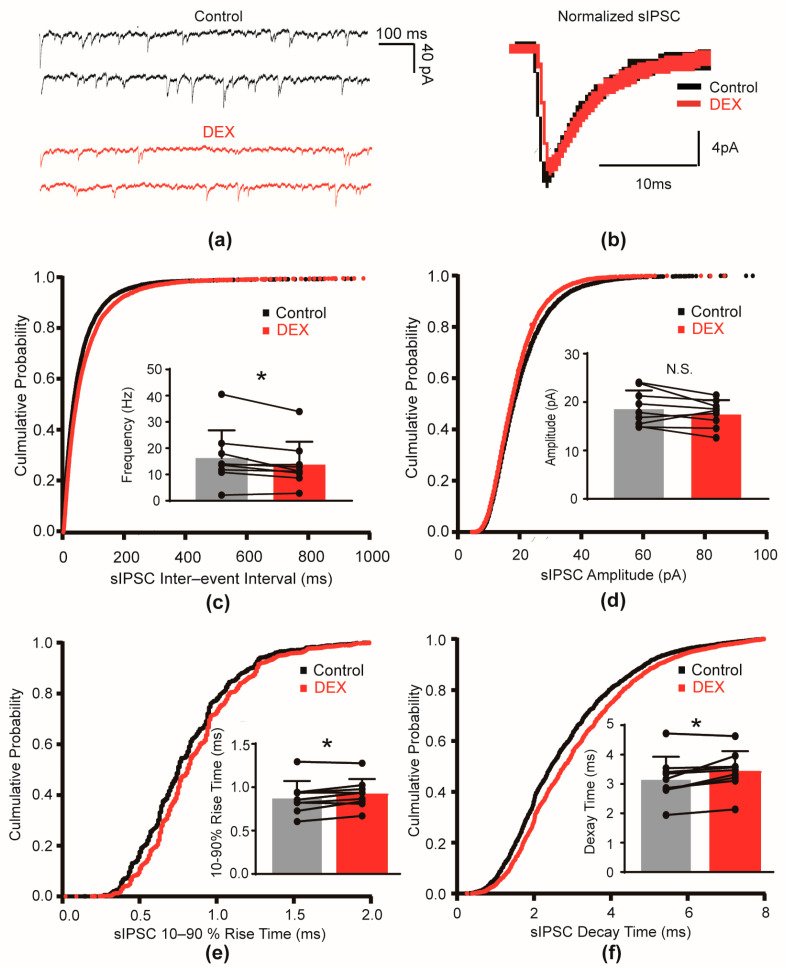
DEX prolonged the decay time of mIPSCs in S1 layer 5 pyramidal cells. (**a**) Representative traces illustrating mIPSCs in the absence versus mIPSCs in the presence of DEX (10 μM); (**b**) representative traces of normalized mIPSCs in the absence of DEX versus mIPSCs in the presence of DEX (10 μM); (**c**,**d**) cumulative probability curve of interevent intervals and amplitudes of mIPSCs before and during DEX application. The inserts in (**c**,**d**) summarize the effect of DEX on the frequency (*p* = 0.263) and amplitude (*p* = 0.779) of mIPSCs, respectively; (**e**,**f**) cumulative probability curve of 10–90% rise time and decay time of mIPSCs before and during application of DEX. The inserts in (**e**,**f**) summarized the 10–90% rise time (*p* = 0.327) and decay time (*p* = 0.025) of mIPSCs. Dots represented the value of each cell. The asterisk (*) indicated *p* < 0.05 and N.S. indicated no significant differences (n = 7). Control indicated a CSF without DEX.

To further investigate the influence of DEX on inhibitory transmission, we examined its effect on miniature inhibitory postsynaptic currents (mIPSCs), which is independent of action potential. Tetrodotoxin (TTX) was used to block action potentials. These mIPSCs were recorded in the presence of TTX. Bath application of 10 μM DEX did not significantly alter either the frequency (control: 14.43 ± 5.92 Hz versus DEX: 15.39 ± 5.96 Hz, *p* = 0.451, Figure 2c) or the amplitude (control: 18.56 ± 9.11 pA versus DEX: 18.38 ± 10.33 pA, *p* = 0.812, Figure 2d) of mIPSCs. These results suggest that DEX did not affect the probability of GABA release, independent of action potential-mediated mechanisms.

However, we found that DEX significantly prolonged the decay time of mIPSCs, similar to its effect on sIPSCs. The mIPSC decay time increased from 2.61 ± 0.74 ms in control conditions to 2.76 ± 0.73 ms upon DEX application (*p* = 0.014, Figure 2f). Conversely, no significant change was observed in the 10–90% rise time of mIPSCs following DEX treatment (control: 0.75 ± 0.16 ms versus DEX: 0.78 ± 0.13 ms, *p* = 0.263, Figure 2e). This further confirmed that DEX primarily modulated the decay phase of GABA-mediated inhibitory currents, indicating a postsynaptic mechanism of action.

### 2.3. DEX Had No Effect on Evoked Inhibitory Postsynaptic Potentials

**Figure 3:**
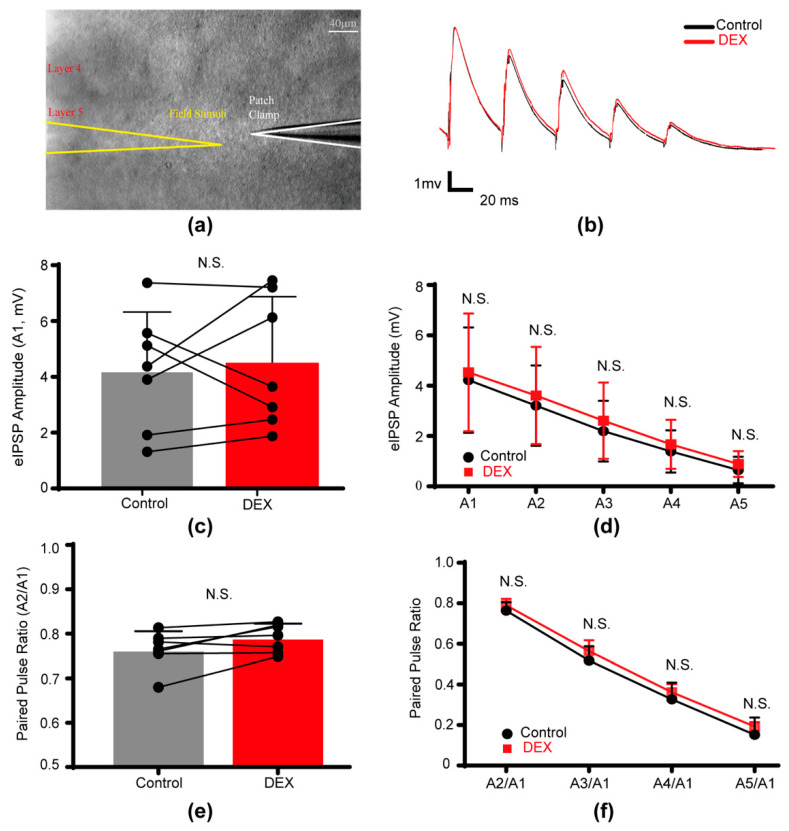
DEX had no effect on evoked inhibitory postsynaptic potentials in pyramidal cells of layer 5 in S1. (**a**) Schematic representation of the evoked IPSP (eIPSP) recording approach; (**b**) average traces of eIPSPs recorded from a neuron under control and DEX conditions; (**c**) summary of the effect of DEX on the amplitude of first eIPSPs (A1, *p* = 0.499); (**d**) effects of DEX on A1, A2, A3, A4, and A5; (**e**) summary of the effect of DEX on the PPR of A2/A1 (*p* = 0.063); (**f**) effects of DEX on PPRA2/A1, PPRA3/A1, PPRA4/A1, PPRA5/A1. Dots in (**c**,**e**) represented the value of each cell. N.S. indicated no significant differences (n = 7). Control indicated a CSF without DEX.

To examine the impact of DEX on action potential-evoked inhibitory neurotransmission, we electrically stimulated layer 5 cells in the same cortical column as the recorded pyramidal neuron and measured evoked inhibitory postsynaptic potentials (eIPSPs) (Figure 3a). We specifically assessed the effects of DEX on the amplitude, 10–90% rise time, and decay time of the first eIPSP, as well as the paired-pulse ratio (PPR).

We found that bath application of 10 μM DEX did not significantly alter the average peak amplitude (control: 4.22 ± 2.10 mV versus DEX: 4.53 ± 2.34 mV, *p* = 0.499, Figure 3c,d, Table S1), 10–90% rise time (control: 3.50 ± 0.86 ms versus DEX: 3.82 ± 0.77 ms, *p* = 0.497), or decay time (control: 28.03 ± 4.08 ms versus DEX: 27.84 ± 5.03 ms, *p* = 0.892) of the first eIPSP. Furthermore, we observed a slight, but non-significant increase in the PPR of A2/A1 (the amplitude ratio of the second to the first eIPSP) following DEX application (control: 0.76 ± 0.04 versus DEX: 0.79 ± 0.03, *p* = 0.063, Figure 3e, Table S1). These findings indicated that DEX did not substantially affect action potential-evoked GABAergic transmission, including both pre- and postsynaptic components.

## 3. Discussion

Our study provided evidence that DEX modulated GABAergic transmission in layer 5 pyramidal neurons of the somatosensory cortex through both pre- and postsynaptic mechanisms. Specifically, DEX reduced spontaneous activity-dependent GABA release in presynaptic terminals while also prolonging the duration of postsynaptic GABA_A_R-mediated inhibitory signals. However, stimuli-evoked GABAergic transmission was not changed by DEX. These findings suggest that the impact of DEX on cortical GABAergic signaling may underlie its known sedative, analgesic, and anesthetic-sparing properties.

Our observation of a reduction in sIPSC frequency, coupled with the absence of a change in mIPSC frequency, strongly suggested that DEX inhibited action potential-dependent GABA release in S1. Our finding was consistent with prior studies [9,10]. The explanation of the differential effects of DEX on action potential-dependent and action potential-independent GABA release onto L5 pyramidal neurons would be that presynaptic a_2A_ adrenoceptors might not affect the synaptic release machinery downstream of the Ca^2+^ influx in GABAergic nerve terminals. Since GABA release relies on action potential-induced Ca^2+^ influx of presynaptic cells [11], DEX-induced inhibition of hyperpolarization-activated cyclic nucleotide-gated (HCN) channels and/or voltage-dependent Ca^2+^ channels may play a role [9,12,13,14]. However, it is noteworthy that some studies have reported an increase in spontaneous GABA release by α_2_-AR activation in other cortical regions [15]. This discrepancy likely reflects the region- and layer-specific nature of adrenergic modulation of GABAergic transmission within the cortex [16,17]. Given the heterogeneity of GABAergic interneurons and the diversity in their laminar organization throughout the cortex [18,19], the heterogeneous distribution of adrenergic receptors on these interneurons and their diverse axon terminals could also lead to varying impacts of α_2_-AR activation on GABAergic signaling across different areas [13]. These results emphasized the complexity of α_2_-AR modulation of GABAergic transmission in the cortex, while our findings on the specific effects of DEX in layer 5 of S1 contributed to understanding the effects of the locus coeruleus–norepinephrine system on cortical synaptic function. Future studies exploring the specific ion channels involved in mediating the DEX-induced decrease in GABA release probability on presynaptic interneurons are needed.

Our findings indicate that DEX exerted significant postsynaptic effects on layer 5 pyramidal neurons, as evidenced by prolonged rise and decay times of sIPSCs and prolonged the rise time of mIPSCs. While the precise mechanism underlying these changes in gating kinetics requires further investigation, one possibility involves the modulation of HCN channels. Activation of α_2_-ARs inhibits HCN channels by reducing cyclic adenosine monophosphate (cAMP) production, a known physiological modulator that facilitates HCN channel opening [20]. A reduction in HCN channel activity may decrease membrane capacitance, thus weakening its ability to attenuate synaptic signals. However, it is essential to recognize that IPSC kinetics are influenced by multiple factors, including the temporal profile of GABA exposure (which is affected by GABA transporter density and the synchrony of presynaptic GABA release), the presence of perisynaptic GABA_A_Rs, intracellular chloride ion concentration, and the intrinsic properties of postsynaptic GABA_A_Rs [21,22,23,24]. Further research is needed to fully elucidate the mechanisms behind DEX-induced prolongation of IPSCs.

The phosphorylation of GABA_A_R subunits through cAMP-dependent pathways is known to modulate GABAergic transmission via regulating receptor endocytosis [25]. While some studies have reported that DEX enhanced GABAergic synaptic activity by increasing the interaction of phosphorylated Akt (p-Akt) with GABA_A_R subunits [26], leading to increased mIPSC frequency, evoked IPSC amplitude, our current results did not reveal a significant change in the amplitude of sIPSCs, mIPSCs, or eIPSPs as a result of DEX treatment. This apparent discrepancy may be attributed to area- and layer-specific distributions of GABA_A_R subunits and their differential regulation by protein kinase A (PKA)-mediated phosphorylation [25]. It is crucial to note that phosphorylation at different sites on GABA_A_R subunits can produce divergent effects [27,28].

We proposed that the observed effects of DEX on cortical GABAergic signaling contributed to DEX-induced changes in neuronal activity within the cortex, thereby underlying some of its clinical features. While the precise molecular and cellular mechanisms of general anesthesia remain incompletely understood, the GABA_A_R is widely recognized as a crucial target for many general anesthetics [29]. Anesthetics like etomidate and propofol enhance chloride conductance by prolonging the decay time and increasing the amplitude of IPSCs through interactions with β+/α− subunit interfaces on synaptic GABARs [30,31]. Given that propofol consumption is significantly reduced as a result of DEX administration [1], we speculate that DEX may synergize with propofol by prolonging the opening time of GABA_A_R channels, allowing for an increased chloride ion outflow. Moreover, DEX-induced alterations in the gating kinetics of GABA_A_Rs may modulate the time window of signal transmission, potentially contributing to neuronal synchronization, which relates to anesthetic-induced loss of consciousness [32]. Indeed, the presence of brain-wide synchronized activity in layer 5 pyramidal neurons of cortex during anesthesia has been proposed as a general principle of anesthetic-induced loss of consciousness [33]. Intriguingly, the present study indicated that DEX did not significantly alter stimulus-evoked inhibitory signals (eIPSPs). It may relate to rapid arousability upon DEX administration.

This study has several limitations. Firstly, we focused our investigation of DEX on a single concentration (10 μM) in order to assess its effects on GABAergic neurotransmission. This concentration was selected based on preliminary experiments and previous literature [14]. Our preliminary dose–response assessment, which examined the effect of varying DEX concentrations (0.2 μM, 10 μM, and 30 μM) on the resting membrane potential of neurons (Supplementary Materials, Figure S1 and Table S2), showed that all three concentrations caused slight depolarizations on average. Notably, this depolarization trend remained consistent in the 10 μM DEX group. Furthermore, the chosen 10 μM concentration is clinically relevant, aligning with the upper range of therapeutically relevant concentrations reported in the literature (0.05 μM to 10 μM) [34,35]. Despite this rationale, we recognize that the dose–response relationship of DEX on signal transduction might differ from the dose–response profile of resting membrane potential studies. Second, our study measured total inhibitory inputs onto layer 5 pyramidal cells, limiting the ability to distinguish between different types of interneurons. Given the diverse GABAergic interneuron innervation of layer 5 pyramidal cells in S1 [36], paired recordings between interneuron subtypes and pyramidal cells would be necessary to clarify the specific effects of DEX on specific GABAergic transmission. Finally, the sample sizes used in our electrophysiological recordings were relatively small, which may have reduced our power to detect significant differences. Although it is common practice to have a sample size of around 10 for paired within-subject comparisons in electrophysiological experiments, especially when using brain slice models [37,38], a larger sample size is still recommended for future studies.

In conclusion, our study demonstrated that DEX exerted a dual effect on GABAergic transmission onto pyramidal cells: decreasing presynaptic spontaneous action potential-dependent GABA release while simultaneously prolonging postsynaptic GABA_A_R responses. However, the stimuli-evoked action potential-dependent GABAergic transmission was not changed during DEX application. The physiological consequences of these simultaneous pre- and postsynaptic modulations warrant further exploration.

## 4. Materials and Methods

### 4.1. Slice Preparation

C57BL/6 *mice* of either sex, which were 15~21 postnatal days old, were intraperitoneally anesthetized with 1% sodium pentobarbital (50 mg/kg) and then decapitated. The parasagittal slices of the S1 with a thickness of 250 μm were cut in high-sucrose solution containing (in mM) 2.5 KCl, 1.25 NaH_2_PO_4_·H_2_O, 26 NaHCO_3_, 10 dextrose, 213 sucrose, 2 MgSO_4_, and 2 CaCl_2_ [39]. Afterwards, slices were immediately transferred to an incubation chamber filled with aCSF (in mM: 126 NaCl, 2.5 KCl, 1.25 NaH_2_PO_4_, 26 NaHCO_3_, 25 dextrose, 2 MgSO_4_, 2 CaCl_2_; 315~325 mOsm, pH = 7.2~7.3) bubbled with 95% O_2_ and 5% CO_2_ at 35.5 °C for 30~40 min. Thereafter, the slices were kept at room temperature before use.

### 4.2. Electrophysiological Recordings

Individual slices were transferred to a submerged recording chamber and perfused with bubbled (95% O_2_ and 5% CO_2_) aCSF at a rate of 2~3 mL/min using a roller pump at 36.5~37.5 °C. An upright fixed-stage motorized microscope BX-51WI (Olympus, Tokyo, Japan) with infrared-differential interference contrast was used. Pyramidal cells in layer 5 of *mouse* S1 were recognized for their morphological and electrical properties (see Supplementary Materials, Figure S2 and Table S3). They were recorded using the whole-cell patch-clamp technique and filled with biocytin (0.2%) for post hoc staining. Recording pipettes with an impedance of 3~7 MΩ were used. DEX was prepared by dissolving the powder in water to create a stock solution (100 µM), which was then diluted to 10 µM.

During the recording of IPSCs and IPSPs, 10 μM 6-cyano-7-nitroquinoxaline-2,3-dione (CNQX) and 50 μM D-2-amino-5-phosphonovalerate (D-AP5) were applied to block glutamatergic transmission. CNQX was dissolved in 1% dimethyl sulfoxide with saline to create a stock solution (10 mM), which was then diluted to the desired working concentration (10 µM) in the extracellular solution. D-AP5 was prepared by dissolving the powder in water to create a stock solution (50 mM), which was then diluted to 50 µM. Meanwhile, a high-Cl-based internal solution was used, containing (in mM) 72 K-gluconate, 71 KCl, 2 MgCl_2_, 10 HEPES, 0.2 EGTA, 2 Na_2_ATP, and 0.2% biocytin (292 mOsm, pH 7.2). Activity-independent mIPSCs were recorded in the presence of 1 μM TTX, a blocker for voltage-gated Na^+^ channels. TTX was dissolved in water to create a stock solution (1 mM), which was then diluted to 1 µM. The IPSCs were recorded at a holding potential of −70 mV.

During the recording of eIPSPs, we placed a stimulation micropipette (inner diameter of about 2 μm) filled with ACSF and connected to the outlet of the ISO-Flex isolated output stimulator (AMPI, Jerusalem, Israel) at the same layer but 150–300 μm from the recorded cell. A paired-pulse protocol with a 50 ms interstimulus interval was performed. The stimulation electrode delivered trains of stimuli (a train of five stimuli, each one lasting 0.1 ms, 50 ms apart, every 10 s, with 30 repetitions) to evoke IPSPs. The stimulus intensity was 1.5 times the minimal pulse intensity, which could elicit a response from the recorded cell. The amplitude, and duration of the evoked IPSPs could be accessed, and paired-pulse ratio (PPR) can be calculated. PPR = amplitude of 2nd IPSP/Amplitude of 1st IPSP. A PPR > 1 suggests enhanced synaptic transmission; a PPR < 1 suggests decreased synaptic transmission.

Voltage or current clamp recordings were performed using a MultiClamp 700B amplifier (Molecular Devices, San Jose, CA, USA). Micro1401-3 or Power1401-3A together with Spike2 (version 8, CED, Cambridge, UK) were used for data acquisition. Voltage and current signals were filtered at 10 and 3 kHz and sampled at 50 kHz. Series resistance (10–25 MΩ) was continuously monitored, and data were discarded if the resistance changed by more than 30%.

### 4.3. Immunohistochemistry

After recording, slices with cells filled with biocytin were fixed in 4% paraformaldehyde for at least two hours. Then, they were rinsed in 0.01 M phosphate-buffered saline (PBS) three times, transferred to 0.5% Triton X-100 for 30 min and then incubated in a blocking solution (5% BSA in PBS) for 1 h at room temperature. Afterwards, slices were incubated with streptavidin (Alexa Fluor 488, Invitrogen, Eugene, CA, USA, 1:2000) for 12 h at 4 °C. Z stack images were acquired with a 40× air objective on a confocal microscope (Nikon A1 plus, Tokyo, Japan) and processed using ImageJ (version 1.52a, National Institutes of Health, Bethesda, MD, USA).

### 4.4. Data Analysis

Synaptic currents and potentials were analyzed using MiniAnalysis software (version 6.0.3, Synaptosoft, Decatur, GA, USA). The IPSC events that occurred within 1 min were calculated. The IPSC events which occurred under control conditions were analyzed during a stable duration just before DEX application, while those under DEX conditions were analyzed during a duration of 4~5 min after the initiation of the DEX application. For eIPSP recording, the DEX data were obtained from 4~5 min after the start of DEX application as well. The PPR of eIPSPs was calculated as the mean amplitude of the second, third, fourth and fifth eIPSPs (A2, A3, A4, A5) divided by that of the first eIPSPs (A1). A kinetic analysis of the postsynaptic inhibitory signals was performed by measuring the 10–90% rise time and the time constant of decay.

### 4.5. Statistical Analysis

We used graphical distribution methods for data normality testing. We used repeated-measures ANOVA tests if they were normally distributed; otherwise, we used a Wilcoxon signed-rank test for paired data obtained before and during DEX treatment of the same neuron. Statistical comparisons and data plotting were performed using SPSS (version 22.0, IBM, Armonk, NY, USA) and GraphPad Prism 7 (version 7, GraphPad Software, San Diego, CA, USA). The group data in the main text and the figures are presented as the mean ± SD. *p* < 0.05 was statistically significant. All raw data and analyzed data are available upon request.

### 4.6. Drugs and Chemicals

Dexmedetomidine, CNQX, and D-AP5 were purchased from R&D Systems (Minneapolis, MN, USA). TTX was purchased from Chengdu Must Biotechnology (Chengdu, China). Additionally, reagents used for slicing solution, aCSF, and internal pipette solution were all purchased from Sangon Biotech (Shanghai, China). Biocytin was purchased from Sigma-Aldrich (Shanghai, China) and streptavidin (Alexa Fluor 488) was purchased from Invitrogen (Eugene, CA, USA).

## Data Availability

Data will be made available upon reasonable request.

## Supplementary Materials

The following supporting information can be downloaded at https://www.mdpi.com/article/10.3390/ijms26051931/s1.

## Abbreviations

The following abbreviations are used in this manuscript:

DEX	Dexmedetomidine
α_2_-AR	α_2_-adrenoceptor
GABA	γ-aminobutyric acid
GABA_A_ receptor	GABA_A_R
S1	Primary somatosensory cortex
sIPSC	Spontaneous inhibitory postsynaptic currents
mIPSC	Miniature inhibitory postsynaptic current
eIPSP	Evoked inhibitory postsynaptic potential
PPR	Paired-pulse ratio
