# Supplementary material for: Dexmedetomidine Reduces Presynaptic γ-Aminobutyric Acid Release and Prolongs Postsynaptic Responses in Layer 5 Pyramidal Neurons in the Primary Somatosensory Cortex of Mice
Source: Int J Mol Sci. 2025 Feb 24;26(5):1931. doi: 10.3390/ijms26051931 (PMC11900034; doi:10.3390/ijms26051931)
Supplement: Supplementary file 1 [file ijms-26-01931-s001.zip › ijms-3420697-supplementary.pdf]

Supplementary materials

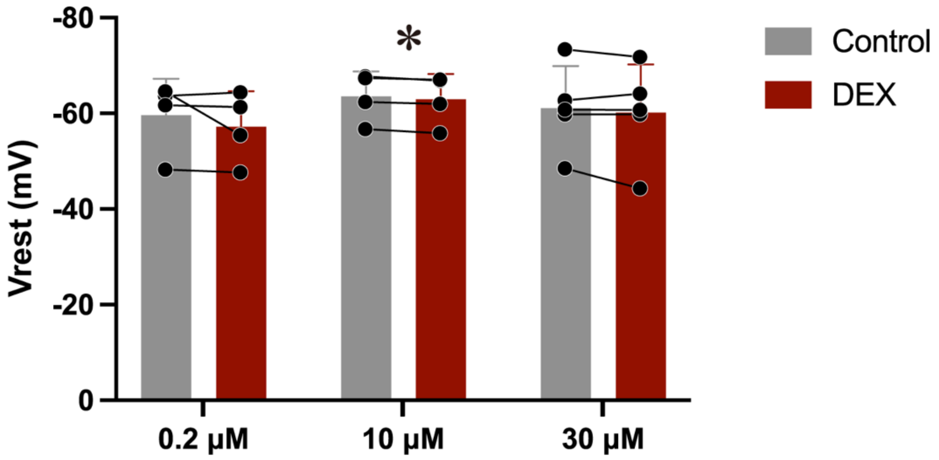

Figure S1. Effects of varying concentrations of DEX on the resting membrane potential of L5 pyramidal cells. DEX of 0.2 μM, 10 μM, and 30 μM were tested. The depolarization induced by 10 μM DEX reached statistical significance, although there was a minimal change was found. The asterisk (*) indicated $p<0.05$. Paired-t test was used.

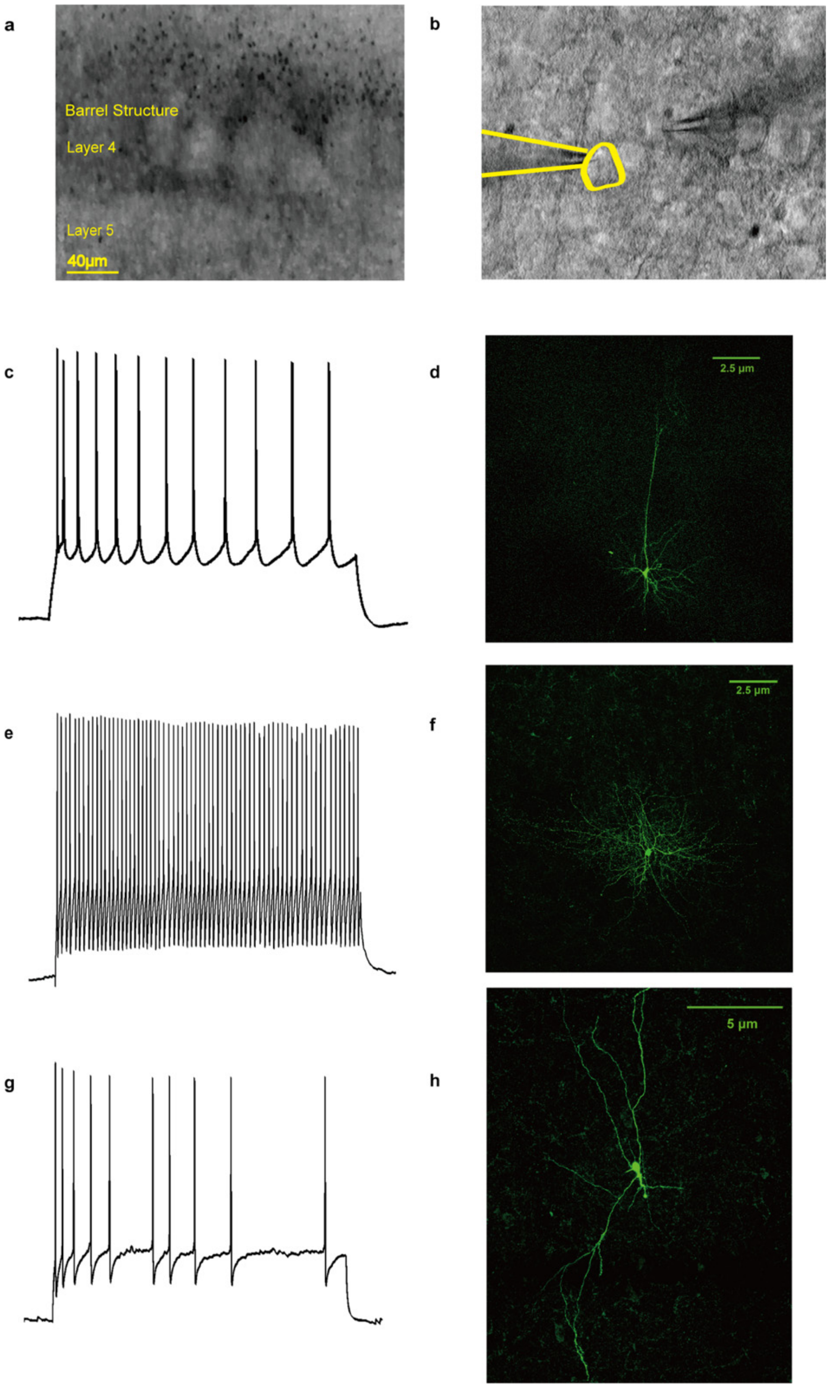

Figure S2. Schematic representation of the identification approach of layer 5 pyramidal cells in the mouse primary somatosensory cortex. **(a)** Somatosensory cortex layer 4 can be seen with light hollow barrel-like structures with narrow dark stripes in between at low magnification. Layer 5 is located directly below barrel-like structures; **(b)** The typical large pyramidal shape of the layer 5 pyramidal cell is shown under a light microscope at high magnification (yellow outline); **(c)** Cortical pyramidal neurons in layer 5 show a regular firing pattern, while the GABAergic interneurons always show fast-spiking firing **(e)** or irregular spiking both with deep afterhyperpolarization **(g)**; **(d)** After patch-clamp recording, biocytin labeling was immunohistochemically processed to permanently stain the neurons. Layer 5 pyramidal cells show a pyramidal-shaped soma and prominent apical dendrites, while GABAergic interneurons generally show large and/or elongated/ovoid somas, and the apical dendrites are not significant **(f,h)**.

Table S1. Amplitudes and PPRs of eIPSPs without and with DEX application

	**Control**	**DEX**	**p value**	**95% Confidence Interval**
A1	4.22±2.10	4.53±2.34	0.499	-1.50, 2.12
A2	3.21±1.60	3.61±1.94	0.524	-1.03, 1.82
A3	2.20±1.21	2.61±1.51	0.379	-0.65, 1.48
A4	1.39±0.85	1.67±0.97	0.382	-0.45, 1.02
A5	0.65±0.53	0.89±0.52	0.257	-0.23, 0.71
PPR A2/A1	0.76±0.04	0.79±0.03	0.063	-0.06, 0.00
PPR A3/A1	0.52±0.07	0.56±0.05	0.057	-0.09, 0.00
PPR A4/A1	0.33±0.08	0.36±0.04	0.158	-0.09, 0.02
PPR A5/A1	0.15±0.09	0.19±0.02	0.157	-0.10, 0.02

A1-A5 indicated first to five amplitudes of eIPSP, which elicited by a train of five stimuli; PPR indicated paired-pulse ratio; CI indicated confidence interval.

Table S2. Effects of varying concentrations of DEX on the resting membrane potential of L5 pyramidal cells

**DEX concentration (uM)**	**Cell numbers**	**Vrest without DEX (mV)**	**Vrest with DEX (mV)**	**Difference**	**p value**
**0.2**	4	-59.62±7.63	-57.25±7.38	2.367839	0.465
**10**	4	-63.59±5.17	-62.96±5.27	0.625447	0.068
**30**	5	-61.09±8.84	-60.20±10.03	0.895675	0.225

Data were presented as the mean ± standard deviation. Wilcoxon signed-rank test was used.

Table S3. Firing properties of L5 pyramidal neurons

**V_{rest} (mV)**	**AMP (pA)**	**R_{in} (MΩ)**	**HW (ms)**	**AHP (mV)**	**Threshold (mV)**
-66.80±6.65	65.04±12.17	199.01±50.76	0.74±0.12	9.66±3.23	-38.87±7.07

Properties of the first action potential (AP) of first spike were analysed from 23 cells. V_{rest}, resting membrane potential; AMP, amplitude; R_{in}, input resistance; HW, AP half-width; AHP, afterhyperpolarization.
